# Leveraging Temporal Down-Sampling Structure and Spatio-Temporal Fusion for Efficient Video Coding †

**DOI:** 10.3390/s26051522

**Published:** 2026-02-28

**Authors:** Keren He, Yufei Gao, Qi Wang, Haixin Wang, Jinjia Zhou

**Affiliations:** Graduate School of Science and Engineering, Hosei University, Tokyo 184-8584, Japan; keren.he.6r@stu.hosei.ac.jp (K.H.); yufei.gao.8e@stu.hosei.ac.jp (Y.G.); qi.wang.7c@stu.hosei.ac.jp (Q.W.); haixin.wang.8v@stu.hosei.ac.jp (H.W.)

**Keywords:** video coding, video enhancement, deep learning, low-bitrate

## Abstract

Down-sampling-based video compression frameworks have shown great potential in improving compression efficiency in modern sensing and imaging systems. However, existing methods ignore critical spatial and temporal redundancy, and treat all frames uniformly during down-sampling. This leads to the loss of important information and impacts compression efficiency. To address these limitations, this paper proposes a temporal down-sampling system, in which only intermediate frames are down-sampled while preserving key frames with high quality for reference. On the decoding side, we employ a frame-recurrent enhancement mechanism to maximize the use of temporal redundancy information. In the fusion of enhancement stage, we design a Multi-scale Temporal-Spatial Attention (MTSA) module. MTSA consists of two components: Multi-Temporal Attention (MTA) and Pyramid Spatial Attention (PSA). MTA performs multi-scale temporal correlation modeling, expanding the receptive field and providing stable cues in compressed regions. PSA integrates local spatial saliency and contextual structure in a progressive and multi-stage manner. Extensive experiments show that our approach achieves consistent BD-rate reductions. Under All-Intra, Low-Delay-P, and Random Access configurations, we observe BD-rate reductions of I, P, and B frames ranging from 14% to 39% compared to VVC, and outperform prior approaches anchored by the standard HEVC.

## 1. Introduction

In the current era of digital information, digital videos serve as an important medium for information exchange and are widely generated by various visual sensing and imaging systems, such as surveillance cameras, mobile devices, and intelligent sensors. Therefore, the demand for high-quality videos are constantly increasing. However, high-quality videos generally contain significantly large amounts of information, requiring extremely high transmission bandwidths, which are often difficult to obtain in current telecommunication systems [1]. Over the past few decades, a series of video coding standards, such as H.264/AVC [2], H.265/HEVC [3], and H.266/VVC [4], have been systematically developed to meet the growing demands of video transmission and storage. In recent years, neural video codecs (NVCs) [5,6,7,8,9,10], which leverage deep neural network (DNN) to model video context, have made remarkable progress, demonstrating higher compression efficiency and showing the potential to surpass traditional coding paradigms. However, spatial and temporal redundancies still remain within video sequences and have yet to be fully eliminated. Moreover, these methods still face challenges such as deployment difficulties, especially in resource-constrained sensing platforms or low-power scenarios.

In contrast, the down-sampling-based strategy provides an alternative method for achieving flexible encoding. It still has good practicality in industrial applications, especially in situations where extremely low bitrate transmission is required but certain semantic or structural information is maintained. Down-sampling-based compression has demonstrated the capability of enhancing coding efficiency, particularly in low-bitrate coding scenarios, for both image coding [11,12] and video coding [13,14,15]. Existing down-sampling-based video coding schemes mainly focus on traditional video codecs such as HEVC and VVC. Our preliminary work [16] has demonstrated its promising application prospects.

With the latest advancements in deep learning technology, down-sampling coding methods that combine DNN and super-resolution (SR) techniques are gaining increasing attention. DNN-based methods have achieved remarkable performance [17,18]. By embedding lost information into low-resolution videos, DNN can efficiently recover frames during the reconstruction of high-resolution videos. In addition, significant progress has been made in image reconstruction using DNN-based SR networks. These methods [14,19,20,21,22,23] reduce data encoding by downsampling before encoding and reconstruct video data by upsampling after decoding, thus enabling high-quality video transmission under low bandwidth conditions. Shen et al. [21] first proposed a down-sampling-based super-resolution video coding framework, which combines classic image super-resolution algorithms. Ho et al. [14,22] further developed this framework and designed a degraded-aware recovery and reconstruction deep neural network (RR-DnCNN). RR-DnCNN uses a deep-learning-based image super-resolution network and introduces degraded-aware techniques to alleviate the distortion problem. However, in current SR-based video reconstruction coding works, most reconstruction networks ignore the correlation between frames in a video sequence and focus only on the reconstruction of individual video frames. Furthermore, these existing methods apply the same downsampling rate to every frame, neglecting the importance of high-quality reference frames, inevitably losing fine details and reducing perceptual quality during the enhancement process.

**Figure 1:**
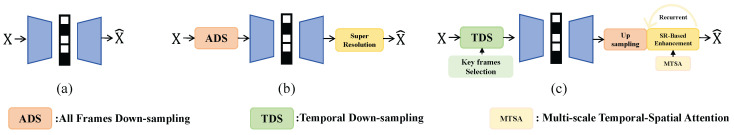
The comparison of our method and existing methods: (**a**) Traditional video codec method. (**b**) Previous down-sampling-based methods. (**c**) Our pipeline with proposed TDS and MTSA model.

Therefore, we propose a novel framework that leverages temporal down-sampling and advanced deep learning techniques. Unlike conventional down-sampling-based coding methods that uniformly down-sample all frames and perform single-pass reconstruction, the proposed framework explicitly differentiates frame importance and integrates progressive multi-frame enhancement. Specifically, our framework significantly reduces the bitrate by partially down-sampling frames, while retaining key frames to exploit inter-frame spatiotemporal correlations. A recurrent enhancement mechanism is then employed to restore spatial details and temporal consistency using a reconstruction network. The key novelty of this work lies in a unified temporal–spatial optimization framework that jointly integrates temporal importance-aware partial down-sampling with iterative frame-recurrent enhancement, which departs from existing uniform DBC strategies. Furthermore, we design a Multi-scale Temporal-Spatial Attention (MTSA) architecture to better exploit inter-frame spatiotemporal information for improved reconstruction and compression performance. Unlike conventional fusion modules, MTSA integrates Multi-Temporal Attention (MTA) and Pyramid Spatial Attention (PSA) to model multi-scale temporal dependencies and hierarchical spatial structures in a structured manner. Through this unified design, the proposed method improves compression efficiency while achieving more accurate reconstruction and better rate–distortion performance, offering a practical and codec-compatible solution for efficient video transmission. Our method demonstrates superior performance in preserving semantic structure and visual fidelity compared to related approaches. Figure 1 illustrates the structural and procedural differences among the three paradigms: (a) traditional video codecs directly encode full-resolution frames and reconstruct them through a standard encoder–decoder pipeline; (b) down-sampling-based methods first reduce spatial resolution before compression and then apply super-resolution for reconstruction; and (c) our proposed framework performs temporal down-sampling with key-frame selection, followed by multi-scale temporal-spatial attention and enhancement to achieve efficient compression while preserving semantic and structural information. In summary, the main contributions of this paper include the following aspects:

1. We propose a temporal down-sampling-based video coding framework (TDS) that selectively preserves high-quality key frames while down-sampling intermediate frames, enabling more effective exploitation of temporal redundancy compared to conventional uniform down-sampling strategies. It can be combined with all the existing coding standards, including HEVC/H.265 and VVC/H.266. And this work is able to reduce the inter-prediction error in encoding.
2. We introduce an iterative SR-based Frame Recurrent enhancement (SRFR) mechanism that progressively propagates high-quality spatial information across frames, improving temporal consistency and reconstruction fidelity beyond single-pass reconstruction approaches.
3. We propose a Multi-scale Temporal-Spatial Attention (MTSA) module that jointly optimizes temporal importance modeling and spatial structure refinement within a unified framework. Specifically, it integrates a Multi-Temporal Attention (MTA) component to model multi-scale temporal correlations and expand the effective temporal receptive field, and a Pyramid Spatial Attention (PSA) component to hierarchically refine structural details and contextual saliency. This joint design allows more effective information propagation across frames and significantly enhances reconstruction fidelity under aggressive down-sampling conditions.

## 2. Related Work

### 2.1. Down-Sampling-Based Coding (DBC)

Down-sampling-based coding (DBC) is a video coding method that reduces data size and transmission bandwidth by down-sampling video frames during the encoding process. Down-sampled frames can be reconstructed using super resolution techniques or other restoration methods to restore their original resolution and quality. In the field of video compression, Shen et al. [21] proposed the DBC framework, which uses super-resolution techniques to restore down-sampled frames to their original resolution. Recently, deep-learning-based super-resolution methods have surpassed traditional approaches in performance. Inspired by these advances, many researchers have further refined the DBC framework. For example, Feng et al. [24] introduced a frame-based DBC system augmented with an enhancement network to remove compression artifacts before the super-resolution stage. Ho et al. [14,22] proposed a Restoration-Reconstruction Deep Neural Network (RR-DnCNN) for end-to-end video image reconstruction based on research on the DBC framework.

More recently, adaptive and learning-assisted strategies have been explored to improve scale control and coding efficiency within DBC-related paradigms. Wang et al. [25] proposed a disparity-aware rescaling learning network for multi-view coding optimization. Cong et al. [26] introduced adaptive sampling strategies to enhance learned video compression efficiency. Lu et al. [27] further extended resampling-based coding within the VVC framework by proposing a learning-based frame-level coding scale control scheme that leverages the Reference Picture Resampling (RPR) mechanism to adaptively adjust coding scales per frame. While these recent studies improve scale adaptation and learning-based control, their designs primarily focus on spatial resolution adjustment or frame-level scale decision, and most approaches treat scale adaptation as an independent per-frame decision without fully exploiting long-range temporal dependencies. This potentially limits the exploitation of inter-frame structural correlations.

Although these approaches demonstrate notable bitrate savings, their designs share several inherent limitations. First, most existing DBC methods apply a uniform spatial down-sampling ratio to all frames, regardless of frame-level importance or temporal significance. Such a strategy ignores the unequal contribution of frames in a video sequence and inevitably discards critical spatial details from key reference frames. Second, these frameworks primarily emphasize spatial restoration while under-exploiting temporal dependencies among frames. As a result, long-range temporal correlations and inter-frame structural consistency are insufficiently preserved. These limitations suggest that a more adaptive down-sampling strategy, particularly one that considers temporal importance, may better balance compression efficiency and reconstruction fidelity. In contrast, we propose a temporal down-sampling video coding framework that adapts the sampling rate to each frame’s significance. By preserving high-quality key frames in full resolution while more aggressively down-sampling less critical frames, our approach maintains essential spatial and temporal details, improves overall visual fidelity, and achieves more efficient bitrate reduction.

### 2.2. Deep Learning Based Video Enhancement

A deep-learning-based enhancement approach leverages deep neural networks to automatically learn complex, hierarchical representations and restore or improve data quality. Its primary advantage lies in its ability to extract and fuse high-level semantic and low-level texture features directly from raw inputs, enabling more accurate detail reconstruction and greater robustness to diverse degradations. As a result, deep learning methods consistently outperform traditional, hand-crafted algorithms in both enhancement effectiveness and computational efficiency. According to recent CNN-based super-resolution achievements [28,29,30,31,32,33,34,35,36,37,38,39,40,41,42,43], transferring the low-size bitstream for high-resolution images/videos is possible. Meanwhile, the use of down-sampling can significantly affect the quality of the decoded video. Fortunately, thanks to the rapid development of SR, enhancement, and deep learning techniques in recent decades, existing works [44,45,46,47,48,49] provide effective SR and Quality Enhancement techniques to restore video quality. And these advanced reconstruction methods can be implemented to improve the quality of decoded videos, which are based on down-sampling [14,19,20,21,22,23]. K. Fischer et al. [19] applied a new module with spatial down-scaling and up-scaling, which combines the VVC codec with machine-learning-based single-image SR algorithms for 4K images. However, this approach only focuses on intra-frame enhancement and does not take advantage of inter-frame information. F. Nasiri et al. [20] proposed a video encoding codec that down-samples and encodes all frames at the encoder and then resizes them at the decoder via super-solution. In Ho’s work [14,22], they proposed an end-to-end restoration-reconstruction deep neural network using the degradation-aware technique, which solves degradation from compression and sub-sampling.

Despite the progress of deep-learning-based enhancement techniques, most existing frameworks treat enhancement as a post-processing step applied independently to each frame or conditioned on limited neighboring frames. Such designs restrict the exploitation of long-range temporal dependencies and fail to construct a global temporal enhancement mechanism. Moreover, enhancement is typically performed in a single forward pass, lacking iterative refinement strategies to progressively improve spatial details and temporal consistency. For example, these methods [14,20,22] leverage inter-frame cues, and they typically perform a one-off enhancement using only a single neighboring frame—thus under-exploiting the rich temporal context available. These observations reveal two fundamental challenges in existing down-sampling-based coding frameworks: (1) the lack of temporal importance-aware sampling strategies, and (2) the absence of iterative, multi-frame enhancement mechanisms capable of progressively refining reconstruction quality. While reducing spatial resolution prior to encoding improves compression efficiency, indiscriminate down-sampling inevitably leads to information loss if critical frames are not adequately preserved. Therefore, a framework that selectively maintains high-quality reference frames while exploiting multi-frame temporal aggregation becomes necessary to better balance compression efficiency and reconstruction fidelity. To overcome this limitation, we propose an iterative enhancement framework that progressively aggregates information from multiple adjacent frames. By repeatedly propagating and fusing inter-frame features, our model maximizes temporal coherence and delivers substantially improved video quality.

### 2.3. Alternative Standard Video Codecs

To enable joint optimization of down-sampling and reconstruction networks, some recent works replace standard codecs with surrogate neural models during training. Jiang et al. [50] and Wei et al. [51] proposed learned proxy codecs to approximate the degradation behavior of traditional codecs, thereby enabling end-to-end training. Chen et al. [52] attempted to better mimic inter/intra coding characteristics, narrowing the modeling gap. However, surrogate codec frameworks generally incur increased computational complexity and training costs, which hinder their applicability in industrial scenarios. These limitations highlight the importance of maintaining compatibility with standard codecs while designing adaptive enhancement strategies that effectively leverage codec-generated context priors.

While surrogate codecs improve optimization flexibility, they introduce approximation gaps between learned models and real-world codec behaviors. In particular, simplified modeling of intra- and inter-frame coding interactions may fail to faithfully capture contextual priors generated by standard codecs. Furthermore, many surrogate approaches primarily focus on intra-frame coding and neglect the complex motion-compensated inter-frame prediction mechanisms used in practical standards such as HEVC and VVC. This discrepancy can limit generalization performance and reduce deployment feasibility. Our codec adopts the standard codec structure, ensuring better compatibility and generalization in real-world applications, and it avoids the limitations of surrogate codec methods in handling contextual information interaction.

## 3. Method

### 3.1. Method Overview

**Figure 2:**
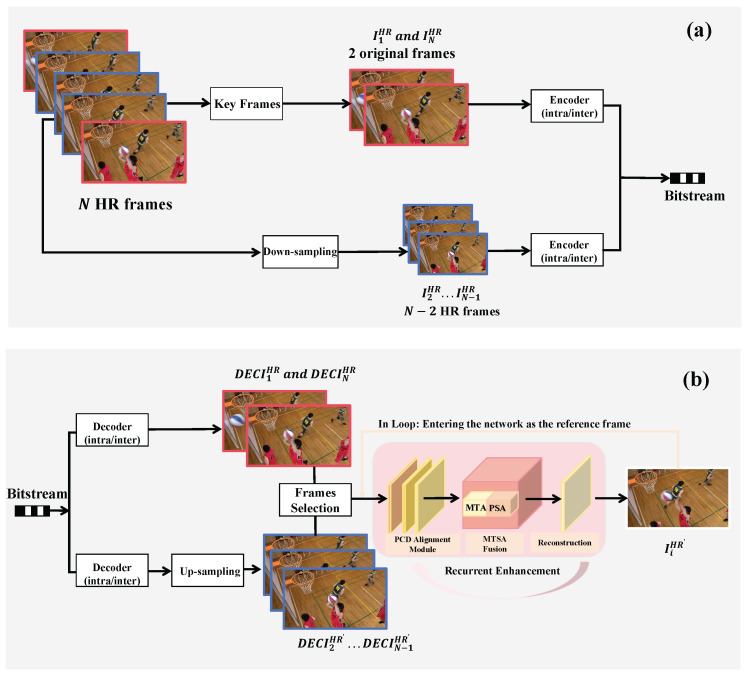
The overall structure of our proposal, (**a**) encoder side (**b**) decoder side.

In this study, our video coding framework consists of three main components: a down-sampling module, a conventional video codec, and a deep-learning-based enhancement network designed to restore high-quality frames from compressed inputs. Initially, we organize the video into Groups of Pictures (GOPs), where each GOP contains an odd number *N* of frames. We utilize FFmpeg with the -sws_flags bicubic option to downsample the intermediate N−2 HR frames using bicubic interpolation to obtain N−2 LR frames. $HR\in R^{H\times W\times 3}$ is down-sampled at the scale of k to have $LR\in R^{H/k\times W/k\times 3}$. The first and last frames are the original HR frames. The video codec is then used to compress all frames. All sequences are down-sampled and compressed into YUV420 format. After decoding the bit stream, up-sampling is applied to the decoded LR frames to obtain HR’ frames with the original size. Finally, the N−2 up-sampled HR’ frames are improved using a deep-learning-based with original frames in a loop. The structure of our proposed method is shown in Figure 2.

### 3.2. Temporal Down-Sampling-Based Coding

**Figure 3:**
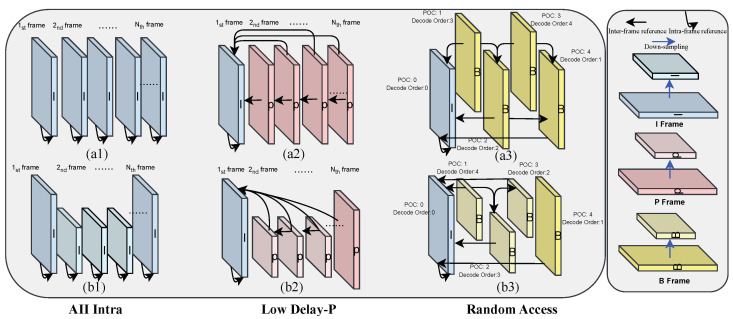
Intra-frame and Inter-frame coding modules in Video Codec: (**a1**/**a2**/**a3**) Original module based on the AI/LDP/RA configurations. (**b1**/**b2**/**b3**) Our proposal module based on the AI/LDP/RA configurations.

On the encoder side, the video can be compressed using VVC / HEVC in the Random Access (RA), Low Delay-P (LDP), and All-Intra (AI) configurations, respectively (InternalBitDepth = 8). Each frame is encoded in an intra-frame mode under the AI configuration. In the LDP configuration, the first picture of the video is coded as an I-frame that uses the intra-frame coding mode, and the inter pictures are coded as P-frames that use the inter-frame coding mode. In the RA configuration, the first frame of the video is encoded as an I-frame using intra-frame coding mode, and the frames between consecutive I-frames are encoded as B-frames using inter-frame coding mode. Referring to the I/P/B-frame information, the RA configuration defines the hierarchical structure between different B-frames. By comparing these three modes, each I-frame of the video can save the majority of data under the AI configuration. However, each P- and B-frame can get interframe information from the other frames under the LDP and RA configurations. So, our proposal modules are designed based on the RA, LDP and AI configurations. The schematic diagrams of these three configuration modules in video coding, as well as our proposed module based on these three configurations, are displayed in Figure 3.

In our work, we compress an *N*-frame video sequence into two groups. First, the first and the *N*-th frames $\left\{I_{1}^{\mathrm{HR}},I_{N}^{\mathrm{HR}}\right\}$ are preserved at full resolution and grouped into a HR sequence for encoding. Second, the down-sampled frames are grouped into a separate LR sequence for encoding. Within each encoded stream, these frames share the standard inter prediction is performed without modification. The decoded picture buffer (DPB), reference list construction, and motion-compensated prediction strictly follow the standard implementation inside each stream.

For our module based on AI configuration, we down-sample $\left\{I_{2},\ldots ,I_{N-1}\right\}$ into an LR video sequence and directly compress the middle frames {$I_{2}^{\mathrm{LR}}$, …, $I_{N-1}^{\mathrm{LR}}$} into a sequence. Under LDP/RA configuration, the intermediate frames, together with the down-sampled version of the first boundary frame, are grouped into a separate LR sequence and encoded as a standard LR stream, and we use the down-sampled frame of the decoded I-frame to decode the bitstream of the middle decoded P/B-frames {${\mathrm{DECI}}_{2}^{\mathrm{LR}}$, …, ${\mathrm{DECI}}_{N-1}^{\mathrm{LR}}$} on the decoder side. The equations used are given by

- Decoded first I-frame and *N*-th I/P/B-frame:
  (1) $$\left({\mathrm{DECI}}_{1}^{\mathrm{HR}},\;{\mathrm{DECI}}_{N}^{\mathrm{HR}}\right)=E_{\mathrm{VVC}}\;\left(I_{1}^{\mathrm{HR}},\;I_{N}^{\mathrm{HR}}\right)$$
- Decoded middle I-frames:
  (2) $$\left({\mathrm{DECI}}_{2}^{\mathrm{LR}},\;\ldots ,\;{\mathrm{DECI}}_{N-1}^{\mathrm{LR}}\right)=E_{\mathrm{VVC}}\;\left(I_{2}^{\mathrm{LR}},\;\ldots ,\;I_{N-1}^{\mathrm{LR}}\right)$$
- Decoded middle P/B-frames:
  (3) $$\left({\mathrm{DECI}}_{2}^{\mathrm{LR}},\;\ldots ,\;{\mathrm{DECI}}_{N-1}^{\mathrm{LR}}\right)=E_{\mathrm{VVC}}\;\left(I_{1}^{\mathrm{LR}},\;\ldots ,\;I_{N-1}^{\mathrm{LR}}\right)$$

### 3.3. Frame-Recurrent Enhancement Module

To effectively refine low-quality frames in a compressed video, we introduce an SR-based Frame Recurrent Enhancement Module that exploits nearby frames as references. Within each group of *N* frames (we assume *N* is odd), the first and last frames are compressed at the original resolution and thus serve as high-quality reference frames, denoted as $\left\{DEC_{1}^{\mathrm{HR}},\;DEC_{N}^{\mathrm{HR}}\right\}$. The remaining frames are compressed at a lower resolution and require enhancement. The corresponding low-quality middle frame is denoted as $DEC_{\mathrm{mid}}^{\mathrm{LR}}$. Let *N* be an odd integer and define the middle index as $\frac{N+1}{2}$.

Since $DEC_{\mathrm{mid}}^{\mathrm{LR}}$ is temporally equidistant from the two reference frames $DEC_{1}^{\mathrm{HR}}$ and $DEC_{N}^{\mathrm{HR}}$, where $G_{\mathrm{enhance}}\left(\cdot \right)$ denotes our enhancement network, this step produces a high-quality middle frame $I_{\mathrm{mid}}^{\mathrm{HR}}$, which is further used as an intermediate reference for the remaining frames in the group. In this way, all frames in the group are recursively enhanced by leveraging both high-quality boundary frames and the refined middle frame. This frame-recurrent scheme enables the network to effectively aggregate temporal information and propagate quality improvements across the entire sequence. We provide more details of this Module in Algorithm 1.
**Algorithm 1** Frame-Recurrent Enhancement**Require:** 
A group of *N* frames, where *N* is an odd integer; let mid=(N+1)/2 denote the middle index. High-quality boundary frames $DEC_{1}^{\mathrm{HR}}$ and $DEC_{N}^{\mathrm{HR}}$; low-quality frames ${\left\{DEC_{i}^{\mathrm{LR}}\right\}}_{i=1}^{N}$; enhancement network $G_{\mathrm{enhance}}\left(\cdot \right)$.**Ensure:** 
Enhanced high-quality frames ${\left\{I_{i}^{\mathrm{HR}}\right\}}_{i=1}^{N}$.  1:*// First enhance the middle frame using both boundary references*  2:$I_{\mathrm{mid}}^{\mathrm{HR}}\leftarrow G_{\mathrm{enhance}}\left(DEC_{1}^{\mathrm{HR}},\;DEC_{N}^{\mathrm{HR}},\;DEC_{\mathrm{mid}}^{\mathrm{LR}}\right)$  3:*// For any frame i* ≠ mid, G_enhance_  *(A, B, C) takes:*  4:*// (i) boundary reference frame A, (ii) enhanced middle frame B, (iii) target low-quality frame C = $DEC_{i}^{\mathrm{LR}}$.*  5:*Case 1: frames before the middle* (1≤i<mid);  6:*// use $DEC_{1}^{\mathrm{HR}}$ and $I_{\mathrm{mid}}^{\mathrm{HR}}$ as references.*  7:**for** i=1 **to** mid−1 **do**  8:      $I_{i}^{\mathrm{HR}}\leftarrow G_{\mathrm{enhance}}\left(DEC_{1}^{\mathrm{HR}},\;I_{\mathrm{mid}}^{\mathrm{HR}},\;DEC_{i}^{\mathrm{LR}}\right)$  9:**end for** 10:*Case 2: frames after the middle* (mid<i≤N); 11:*// use $DEC_{N}^{\mathrm{HR}}$ and $I_{\mathrm{mid}}^{\mathrm{HR}}$ as references.* 12:**for** i=mid+1 **to** *N* **do** 13:      $I_{i}^{\mathrm{HR}}\leftarrow G_{\mathrm{enhance}}\left(DEC_{N}^{\mathrm{HR}},\;I_{\mathrm{mid}}^{\mathrm{HR}},\;DEC_{i}^{\mathrm{LR}}\right)$ 14:**end for**

Previous works such as TDAN [45] and EDVR [46] have emphasized the critical importance of accurately aligning both adjacent frames and reference frames. Precise alignment not only enables the network to better exploit inter-frame information but also significantly contributes to generating high-quality reconstructions. By capturing and integrating temporal dependencies, such alignment mechanisms have proven highly effective in video restoration and enhancement tasks. Motivated by these findings, our enhancement module adopts the EDVR framework [46] as the backbone. In our recurrent enhancement framework, the reference frames and the middle frame must share the same spatial resolution, which necessitates maintaining both the input and output of the network in the high-resolution domain. To satisfy this requirement, we modify the baseline architecture accordingly. Specifically, we remove the explicit up/down-sampling modules in the original EDVR and adapt its alignment and feature fusion components to operate in an HR-to-HR manner. We then retrain the network so that the objective of $G_{\mathrm{enhance}}$ shifts from increasing spatial resolution to focusing on suppressing compression artifacts, restoring fine details, and enhancing temporal coherence. It should be noted that EDVR itself provides an HR-to-HR deblurring configuration, whose task setting is conceptually closer to our enhancement pipeline. Therefore, in the experimental section, we further compare our enhancement network with the EDVR deblurring mode to validate the effectiveness, robustness, and generalizability of our design under the same HR-to-HR setting.

Unlike standard VSR settings, where all input frames are low-resolution and alignment is naturally conducted in the LR domain, our framework intentionally preserves boundary frames at full high-resolution while only intermediate frames are down-sampled for compression efficiency. The preserved HR boundary frames serve as high-fidelity structural references to guide the reconstruction of intermediate frames. If alignment were performed in the LR domain, the HR boundary frames would first need to be down-sampled to match the resolution of intermediate frames. This operation would inevitably discard high-frequency structural details that our framework is explicitly designed to preserve. Such a design would weaken the role of boundary frames as high-quality temporal anchors and contradict the core objective of maintaining reference fidelity. Therefore, in our framework, alignment is performed in the unified HR feature domain. The decoded intermediate frames are first restored to the target HR resolution, and spatio-temporal alignment is then conducted between HR feature representations. This design ensures that the structural information retained in the preserved boundary frames can be fully exploited during feature fusion and enhancement.

### 3.4. Multi-Scale Temporal-Spatial Attention Fusion

**Figure 4:**
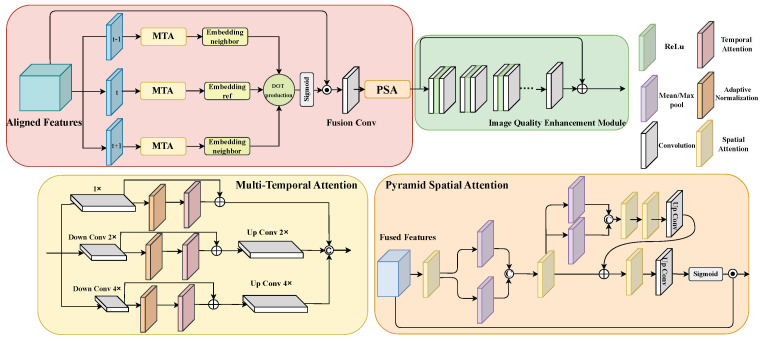
The figure shows the network structure of the frame recurrent enhancement module. The structure contains the MTSA (MTA + PSA) module and the image enhancement module in our proposal. MTA: Multi-Temporal Attention; PSA: Pyramid Spatial Attention.

To better exploit temporal dependencies and spatial details, we propose a Multi-Scale Spatio-Temporal Attention Fusion (MTSA) module, illustrated in Figure 4. MTSA consists of two components: Multi-Temporal Attention (MTA) and Pyramid Spatial Attention (PSA). In order to better compute frame similarity in an embedding space, the MTA module processes the input on three scales—full, 2× downsampled, and 4× downsampled. Each scale undergoes adaptive normalization, temporal attention, and a residual update, after which all branches are upsampled and fused. This design captures both fine textures and large-motion cues. The PSA module further refines the fused features using a three-stage spatial reweighting process. It combines basic spatial attention with pooled feature descriptors from mean- and max-pooling, followed by upconvolution and an additional attention layer. A final learned mask reweights the features, enhancing spatial selectivity. Together, MTA improves inter-frame coherence, while PSA enriches spatial context, resulting in greater robustness to compression artifacts.

(a) Multi-Temporal Attention: The MTA module enhances inter-frame reasoning by aggregating information from neighboring frames at multiple spatial scales. Let ${\left\{F_{\tau }\right\}}_{\tau \in N(t)}$ denote the features of the current frame *t* and its temporal neighbors, where $F_{\tau }\in R^{C\times H\times W}$, and where ${\mathrm{DS}}_{s}\left(\cdot \right)$ denotes spatial downsampling. MTA processes these features at three scales—full resolution, 2× downsampled, and 4× downsampled—to jointly capture fine textures and large-motion cues:

(4) $$X_{\tau }^{\left(0\right)}=F_{\tau },\;X_{\tau }^{\left(1\right)}={\mathrm{DS}}_{2}\left(F_{\tau }\right),\;X_{\tau }^{\left(2\right)}={\mathrm{DS}}_{4}\left(F_{\tau }\right)$$

For each scale s∈{0,2,4}, we first apply adaptive normalization to stabilize the feature distribution. Specifically, AdaNorm refers to an adaptive Layer Normalization mechanism that normalizes channel-wise statistics and modulates feature responses before temporal aggregation. It serves to improve optimization stability rather than enlarging receptive field size. The normalized feature is then fed into a temporal attention module ${\mathrm{TA}}^{\left(s\right)}\left(\cdot \right)$, which computes frame-wise correlations in an embedding space and outputs an attention-refined feature. In our implementation, ${\mathrm{TA}}^{\left(s\right)}\left(\cdot \right)$ adopts standard multi-head scaled dot-product attention along the temporal dimension. For each spatial location, features from different time steps are projected into a embedding space (with embedding dimension equal to the channel dimension of the corresponding scale) via learnable linear projections, and temporal similarity is computed using dot-product operations. The attention weights are then applied to aggregate multi-frame information. To preserve the original content while injecting temporal cues, MTA employs a residual connection between the input of the attention block and its output:

(5) $$X_{t}^{\left(s\right)}=X^{\left(s\right)}+{\mathrm{TA}}^{\left(s\right)}\;\left(\mathrm{AdaNorm}\left(X^{\left(s\right)}\right)\right)$$

Finally, all scales are upsampled back to the original resolution and fused to form the output of MTA:

(6) $${\hat{X}}_{t}^{\left(s\right)}=\mathrm{UpConv}\left(X_{t}^{\left(s\right)}\right),$$

(7) $$F_{\mathrm{MTA}}=\mathrm{concat}\left({\hat{X}}_{t}^{\left(0\right)},\;{\hat{X}}_{t}^{\left(2\right)},\;{\hat{X}}_{t}^{\left(4\right)}\right)$$

where UpConv(·) denotes the corresponding upsampling operation and Concat(·) is implemented by feature concatenation. The multi-scale design is introduced to explicitly model temporal correlation at complementary spatial resolutions. Coarse-scale branches facilitate stable modeling of large motion displacement and heavily compressed regions, while the full-resolution branch preserves fine-grained structural details. Although the multi-scale branches introduce moderate parameter growth, the performance gain primarily arises from structured multi-scale temporal correlation modeling rather than capacity enlargement alone. In this way, the full-resolution branch focuses on fine details, while the lower-resolution branches provide more stable cues for large motions and heavily compressed regions, and their fusion yields a temporally coherent and motion-aware representation for subsequent reconstruction.

(b) Pyramid Spatial Attention: The PSA module refines the fused features using a hierarchical, multi-branch design that progressively enlarges the receptive field and enhances spatial selectivity. Given the input feature $F_{\mathrm{Fusion}}$, this module first applies a spatial-attention block SA and then aggregates complementary global statistics through max-pooling and mean-pooling paths. The pooled descriptors are concatenated and fed into another spatial-attention block, which again produces two global descriptors through max-pooling and mean-pooling. After concatenation and an up-convolution, the result is added to the original feature via a residual connection. Finally, a third spatial-attention block and an up-convolution generate a spatial mask, which is activated by a sigmoid function and used to reweight the input feature map. This pyramid design enables PSA to integrate local spatial saliency, mid-level context, and global statistical structure in a progressive and multi-stage manner. The PSA module operates according to the following equations:

(8) $$\begin{array}{cc} S_{1} & ={\mathrm{SA}}_{1}\left(F_{\mathrm{Fusion}}\right),\\ C_{1} & =\mathrm{Concat}\left(\mathrm{MaxPool}\left(S_{1}\right),\mathrm{MeanPool}\left(S_{1}\right)\right),\\ S_{2} & ={\mathrm{SA}}_{2}\left(C_{1}\right) \end{array}$$

(9) $$R=F_{\mathrm{Fusion}}+\mathrm{UpConv}\;\left(\mathrm{Concat}\left(\mathrm{MaxPool}\left(S_{2}\right),\mathrm{MeanPool}\left(S_{2}\right)\right)\right)$$

(10) $$F_{\mathrm{PSA}}=F_{\mathrm{Fusion}}⊙\sigma \;\left(\mathrm{UpConv}\;\left({\mathrm{SA}}_{3}\left(R\right)\right)\right)$$

Compared to the original EDVR fusion module, MTSA offers three key advantages. First, it performs multi-scale temporal correlation modeling, whereas EDVR computes temporal attention only at a single scale. Second, the multi-scale structure expands the receptive field and provides more stable cues in heavily compressed regions, enabling more reliable texture and structure recovery. Finally, the alignment of multi-scale features improves temporal consistency and reduces flickering, producing smoother and more coherent video results.

(c) Image Quality Enhancement Module: To obtain better reconstruction results, we improve the original reconstruction module. We use a new image quality enhancement (IQEM) module to replace the existing EDVR model. This new model is designed to provide independent and more optimized image quality enhancement capabilities. Through these improvements, we are able to achieve enhanced performance in image processing. For the IQEM module, the main idea of this module is to fully explore important information from fused feature maps and take advantage of residual learning [53,54,55] to generate the enhanced frame. We use a non-linear mapping function $F_{\mathrm{QE}}$ to predict the residual of fused feature maps. And this enhancement module consists of many convolutional layers; each layer employs C2 convolutional filters and uses ReLU as the activation function. This structure enables the network to progressively refine the fused representation and predict an enhanced residual that is then added to the reference frame to obtain the final output.

(d) Loss Function: We adopt the Charbonnier loss function as our final loss, which is a differentiable variant of the L1 loss and is widely used in image restoration and video enhancement tasks due to its robustness to outliers. The loss is defined as follows:

(11) $$L=\sqrt{|I_{i}^{{\mathrm{HR}}^{\prime }}-I_{i}^{\mathrm{HR}}{|}^{2}+\epsilon^{2}}$$

where $I_{i}^{{\mathrm{HR}}^{\prime }}$ denotes the predicted output frame, and $I_{i}^{\mathrm{HR}}$ is the corresponding ground-truth frame. In our experiments, we set ϵ to $1\times 10^{-3}$ following common practice.

## 4. Experiments

### 4.1. Dataset Preparation

For the train dataset, we choose 11 uncompressed videos containing 3300 frames from the SJTU sequences in UHD [56]. The training sequences are down-scaled from 3840 × 2160 to 1920 × 1080 for HR and down-scaled from 1920 × 1080 using bicubic interpolation for LR. We also select additional Class B sequences of 1920 × 1080 size for training, such as BlueSky, Pedestrian, and RushHour. Our work is based on the HEVC/VVC Test Model. Since our scheme requires encoding the 4× down-sampled sequences, we crop the train and test sequences with resolutions of 1920 × 1080 to 1920 × 1072 to satisfy the module training and the coding unit (CU) requirement. The same cropped YUV sequences and identical boundary handling settings were used for both anchor codecs and the proposed framework to ensure strict fairness in RD comparison.

To evaluate our proposed framework, we prepared a custom dataset tailored to our architecture. Specifically, each source YUV video was split into multiple segments, each consisting of *N* consecutive frames using FFmpeg 2.8.22. For each *N*-frame group, the first and last frames were extracted to form one YUV file, while the middle N−2 frames were grouped into another YUV file. To simulate temporal down-sampling, the middle-frame YUV files were spatially downsampled by factors of 2 and 4, respectively, resulting in LR sequences. These LR sequences and their corresponding first/last frames were then compressed using standard video codecs—HEVC and VVC. After compression and decompression, all decoded YUV files were converted to individual PNG images. The YUV-to-RGB conversion for PNG generation strictly follows the BT.709 limited-range specification with consistent transfer characteristics and 8-bit precision. The LR middle frames were subsequently upscaled back to their original resolution to match the frame size for model training. Thus, each group produces a complete training sample.

**Training details:** The experiments of our framework are implemented with Python 3.7 and PyTorch 1.3.0 on Ubuntu 16.04. The training was conducted on NVIDIA GeForce RTX 2080 Ti GPUs (NVIDIA Corporation, Santa Clara, CA, USA). In the training of this network, the scale factor is set to 2 and 4 while training, respectively. We retrain the enhanced model with odd frames and HR. We adopt the Adam [57] optimizer with an initial learning rate of 0.0004 and set the coefficients to $\beta_{1}=0.9$, $\beta_{2}=0.99$, and the weight decay to 0. During training, the size of the ground truth patch is set to 256 × 256. Data augmentation strategies such as horizontal flipping and rotation are applied to improve generalization, and the batch size per GPU is set to 4. This configuration facilitates stable convergence during training. This work has two stages of training:

(a) First stage: We jointly train the PCD Alignment module, baseline backbone network, and PSA module for 600,000 iterations. The objective of this stage is to establish stable cross-frame feature alignment and hierarchical spatial feature representation.

(b) Second stage: The parameters of the PCD Alignment module are frozen to maintain stable feature alignment, while the parameters of the baseline modules are ignored. We focus on training the PSA module, MTA module, and Image Quality Enhancement module for another 600,000 iterations. Furthermore, we perform partial fine-tuning of the higher layers of the PCD module in the later phase of training, thereby enhancing the model’s feature robustness and generalization capability under high-compression conditions.

**Test details:** For evaluation, we select representative sequences from Class B (1920 × 1080) Class C (832 × 480), and Class E (1280 × 720). Specifically, some sequences such as Cactus, BQMall, BQSquare, and Fourpeople are used to assess the performance of our proposed framework.

### 4.2. BD-Rate and BD-PSNR Evaluation Metrics

To quantitatively evaluate the rate–distortion (RD) performance of different coding schemes, we adopt the Bjøntegaard Delta (BD) metrics [58], which are widely used in video coding research.

Given four RD points obtained under different QP settings, a third-order polynomial is used to interpolate the RD curves in the logarithmic bitrate domain. Let r=log(R) denote the logarithmic bitrate and *D* denote distortion measured in PSNR. The BD-PSNR is defined as the average PSNR difference over a common logarithmic bitrate interval $\left[r_{L},r_{H}\right]$:

(12) $$\mathrm{BD}-\mathrm{PSNR}=\frac{1}{r_{H}-r_{L}}\int_{r_{L}}^{r_{H}}\left(D_{2}\left(r\right)-D_{1}\left(r\right)\right)dr,$$

where $D_{1}\left(r\right)$ and $D_{2}\left(r\right)$ denote the interpolated distortion functions of the anchor method and the proposed method, respectively.

Similarly, the BD-Rate is defined as the average logarithmic bitrate difference over a common distortion interval $\left[D_{L},D_{H}\right]$:

(13) $$\mathrm{BD}-\mathrm{Rate}=\frac{1}{D_{H}-D_{L}}\int_{D_{L}}^{D_{H}}\left(r_{2}\left(D\right)-r_{1}\left(D\right)\right)dD,$$

where $r_{1}\left(D\right)$ and $r_{2}\left(D\right)$ represent the interpolated logarithmic bitrate functions corresponding to the anchor method and the proposed method, respectively.

In video coding evaluation, BD-Rate reflects the average bitrate saving at equivalent visual quality, while BD-PSNR reflects the average quality improvement at equivalent bitrate. Therefore, a negative BD-Rate indicates improved compression efficiency, and a positive BD-PSNR indicates enhanced reconstruction quality.

### 4.3. Results Based on VVC

In this section, we evaluate our model on HEVC test sequences, with the reduction in BD-Rate and BD-PSNR anchored by the standard codec VVC as metrics.

**Figure 5:**
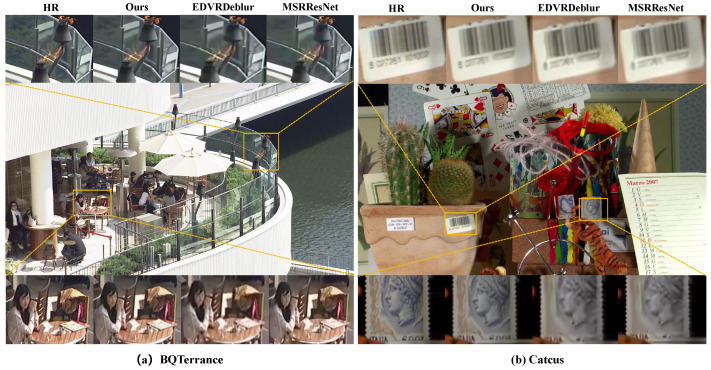
Subjective comparison between the previous work EDVRDeblur, MSRResNet and our method in PSNR (dB)/SSIM. The bold values represent the best performance. (**a**) BQTerrance: Method (PSNR/SSIM); Ours (**28.22/0.8360**); EDVRDeblur (24.96/0.7324); MSRResNet (25.87/0.7552). (**b**) Catcus: Ours (**29.91/0.7942**); EDVRDeblur (27.39/0.7170); MSRResNet (27.69/0.7221).

(a) BD-Rate Reduction: To further demonstrate the generality and practical effectiveness of our approach, we additionally conduct experiments using 2× down-sampling, which better preserves spatial fidelity. Table 1 presents the BD-Rate and BD-PSNR comparison between the standard VVC and our proposed method, evaluated on per-class and per-sequence basis using Low Delay P, Random Access, and All Intra configurations. The performance is computed separately for P-frames, B-frames, and I-frames, respectively. The average BD-Rate reductions are 25.17%, 27.20%, and 24.88%, respectively, with BD-PSNR gains ranging from 0.69 to 0.83 dB, demonstrating strong performance across various frame types and content complexities. To further benchmark our method, we compare it with a recent related work Wu [59], under the AI configuration with 2× downsampling. As shown in Table 2, our method consistently outperforms both BD-Rate and BD-PSNR in all test sequences.

(b) Subjective Quality: Furthermore, we also compare the work on two test sequences BQTerrance and Catcus with resolution 1920 × 1080 both objectively (PSNR/SSIM) and subjectively. As an experimental result, our method shows clearer edges, shapes such as text and lines, compared to the MSRResNet [60] and EDVR Deblur work, as illustrated in Figure 5. Regarding restoration at low resolution, our method stably outperforms its baseline work on the test sequence. Our work provides a higher PSNR as 28.22 dB, 29.91 dB, and SSIM as 0.836, 0.7942 in LDP configuration, respectively. Overall, the results clearly demonstrate that our method offers a compelling trade-off between bitrate and reconstruction quality, making it a practical choice for efficient video transmission with minimal visual degradation.

As shown in Table 1, the proposed method achieves consistent BD-Rate reductions under all three coding configurations. Notably, the gain under the RA configuration is the most significant, reaching an average reduction of 27.20%. This phenomenon can be reasonably interpreted from a temporal dependency perspective. In the RA configuration, hierarchical B-frames rely on multiple reference frames and exhibit stronger temporal prediction dependency compared to LDP and AI. Since the accuracy of reference frames directly affects the prediction residual, preserving high-quality boundary frames and enhancing intermediate frames through recurrent propagation helps stabilize temporal anchors and improve prediction accuracy. This mechanism tends to reduce the residual variance in inter-frame coding. From a rate–distortion standpoint, lower residual variance generally corresponds to reduced entropy in motion-compensated prediction, which leads to improved bitrate efficiency. This provides a plausible explanation for why the RA configuration exhibits slightly larger BD-Rate gains compared to LDP and AI.

### 4.4. Results Based on HEVC

To validate the effectiveness of our model, it is necessary to compare it with other relevant network models. However, research based on the VVC standard is relatively scarce at present, with most related works based on the HEVC standard. Therefore, to facilitate an effective comparison of our model with these HEVC-based works, we retrain our network to adapt to HEVC and make appropriate modifications to the model structure. These adjustments not only enhance the versatility of our model but also enable a more direct comparison with current mainstream technologies, thereby accurately showcasing the advantages of our model. In this section, we compare our method with state-of-the-art methods DCTIF [61], Li [62], Li + CNN [63], Son [64], Jiang [50], Wei [51], Chen [52] and Wu [59]. For results, we cite the performance results from the original paper [59]. The work [59] retrained the methods of Jiang [50] and Chen [52] and performed a unified evaluation on the HM16.5 platform instead of the libx265 platform used in their original paper, in order to ensure the consistency of the experimental platform.

(a) BD-Rate Reduction: The general comparisons of the test sequences are shown in Table 3. The results of the experiment demonstrate the effectiveness and robustness of our network. We refine our initial approach by using original-size frames to enhance the 2× downsampled intermediate frames. Table 3 presents an overall comparison of the test sequences, showcasing the performance of different methods under identical testing conditions. The experimental results further validate the effectiveness and robustness of our proposed network. Specifically, our method achieves an average BD-rate reduction of 29.355% (without MTSA) and 31.034% (with MTSA), demonstrating a significant advantage over other approaches. Among all the compared methods, our approach achieves the best performance, and the second highest result is the latest work [59].

To further verify the effectiveness of our proposed module, we introduce a new scheme when compared against RR-DnCNN [22] and RR-DnCNN v2.0 [14]. In this scheme, we employ compressed 2× downsampled first and last frames to enhance the compressed and 4× downsampled intermediate frames, thereby further improving the quality of the reference frames. Table 4 shows the objective comparison between our proposed method and RR-DnCNN, RR-DnCNN V2.0 using LDP and RA based on HM16.5 in QP = 32, 37, 42, 47. This work outperforms the other two works in the average BD-rate, BD-psnr. The table shows the results of the calculations for all frames under GOP = 5. Our method achieves an average BD-rate reduction of 10.694% and a PSNR gain of 0.287 dB over RR-DnCNN under the LDP configuration. Compared with RR-DnCNN v2.0, our method achieves a BD-rate reduction of 6.934% and a BD-PSNR gain of 0.185 dB on average under LDP. Under the RA configuration, our method achieves a BD-rate reduction of 7.279% and a PSNR gain of 0.205 dB compared with RR-DnCNN. Compared with RR-DnCNN v2.0, our method achieves a BD-rate reduction of 2.795% while maintaining a comparable PSNR level, with an average BD-PSNR gain of 0.073 dB.

From Table 3, it can be observed that sequences with rich textures and complex structures (e.g., PartyScene and BQTerrace) achieve relatively larger BD-rate reductions. Under the AI configuration, where frames are encoded independently without temporal prediction, performance improvements primarily stem from enhanced spatial reconstruction capability. Sequences containing dense edges, repetitive patterns, and abundant details are more vulnerable to information loss during spatial down-sampling. Conventional reconstruction methods often introduce over-smoothing artifacts, leading to noticeable degradation of fine structures. The proposed framework alleviates this issue by leveraging preserved high-resolution information to guide the reconstruction of down-sampled frames, thereby better maintaining structural consistency and recovering fine details. As a result, the rate–distortion advantage becomes more pronounced in texture-rich sequences.

**Figure 6:**
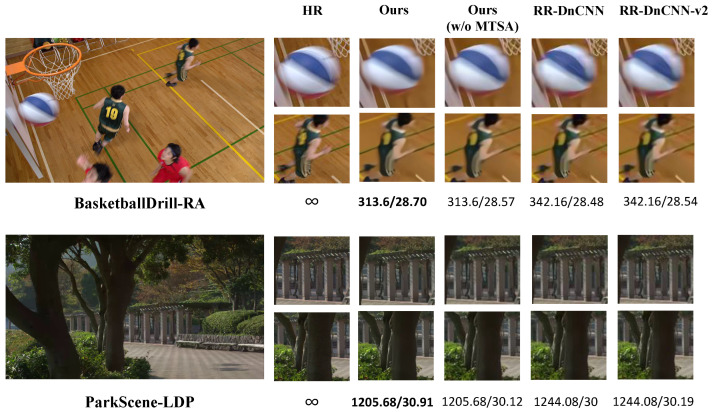
Subjective comparison between our work, RR-DnCNN and RR-DnCNN v2.0 under QP = 37. The measurement information for each method on each video as Bit-rate (kbps)/PSNR (dB). The bold values represent the best performance: (1) BasketballDrill; (2) ParkScene.

(b) Subjective Quality: We compare our method with two methods by RA/LDP configurations as shown in Figure 6; it performs a subjective comparison between our method and other two works in an approximate bit rate condition (QP = 37) in two sequences based on HEVC 16.5. Additionally, we measure the bitrate (kbps)/PSNR (dB) for each method in every compared video. For the BasketballDrill-RA scene, while maintaining good visual quality, our method keeps the bit-rate at 313.6 kbps and achieves a PSNR of 28.70 dB, which is the best performance among all the methods in this scene. In the ParkScene-LDP scene, our method achieves a PSNR of 30.91 dB at a bitrate of 1205.68 kbps; the version after removing MTSA reaches 30.12 dB. For comparison, RR-DnCNN and RR-DnCNN-v2 have bitrates of 1244.08 kbps, with PSNRs of 30 dB and 30.19 dB, respectively. Visual comparisons show that our method maintains sharper details in textured areas such as leaves and pillars, resulting in an overall look closer to the original HR frame.

(c) Model Complexity: In this section, we evaluate the parameter size, the running memory GPU, and BD-rate value across six representative methods, which are presented in Table 5. As shown in this table, Li [62] has the smallest number of parameters (0.396 M) but also achieves the lowest reduction in the BD-rate (−5.15%). Li + CNN [63] slightly increases model size to 0.693 M and reduces GPU memory consumption to 1207 M, leading to an improved BD-rate of −7.11%. Methods with larger network capacities, such as Chen [52] (2.662 M, −12.99%) and Jiang [50] (40.954 M, −10.461%), require significantly higher GPU memory (7398 M and 4306 M, respectively) to achieve better compression performance. Wu [59] further improves the BD-rate to −19.754% with the parameters 1.862 M. In comparison, our model without MTSA reaches a BD-rate of −29.36% with only 2232 M GPU memory. With the proposed MTSA module enabled, our full model achieves the best BD-rate performance of −31.03% using 29.3 M parameters and 4084 M GPU memory, demonstrating a superior trade-off between parameter size, memory consumption, and compression efficiency.

### 4.5. Ablation Study

In this section, we present several ablation experiments to evaluate the effectiveness of the proposed model.

(a) The effectiveness of the proposed modules: First, we conduct extensive ablation studies in three configurations. The results in Table 6 are calculated based on the average bitrate of I, B, and P frames, following the default GOP structure of the VTM encoder. As shown in Table 6, our baseline model TDS + SRFR already achieves notable coding efficiency. When the MTSA module is integrated, the performance improves significantly across all configurations. In particular, the BD-Rate is reduced from 20.779% to 25.165% on LDP, from 18.882% to 27.198% on RA and from 20.372% to 24.884% on AI. Meanwhile, BD-PSNR also shows consistent gains, indicating that MTSA contributes both to bitrate reduction and reconstruction quality. Additionally, the performance using AI configuration based on HEVC is summarized in Table 7. The model with MTSA (TDS + SRFR + MTSA) achieves the best result with a BD-Rate reduction of 31.034% and a more stable BD-PSNR of 1.157, confirming the robustness of MTSA in intra-frame coding scenarios. Table 8 presents an objective comparison between our method and two baselines, RR-DnCNN and RR-DnCNN-V2, using LDP and RA configurations based on HEVC. Our method (TDS + SRFR + MTSA) consistently outperforms both baselines across all settings, achieving the best BD-PSNR and BD-Rate. For example, under LDP, it improves the BD-Rate reduction by up to 10.694% compared to RR-DnCNN and 6.934% compared to RR-DnCNN-V2. Under RA, the gains are also evident, with improvements in the BD-Rate of 7.279% and 2.795%, respectively.

**Figure 7:**
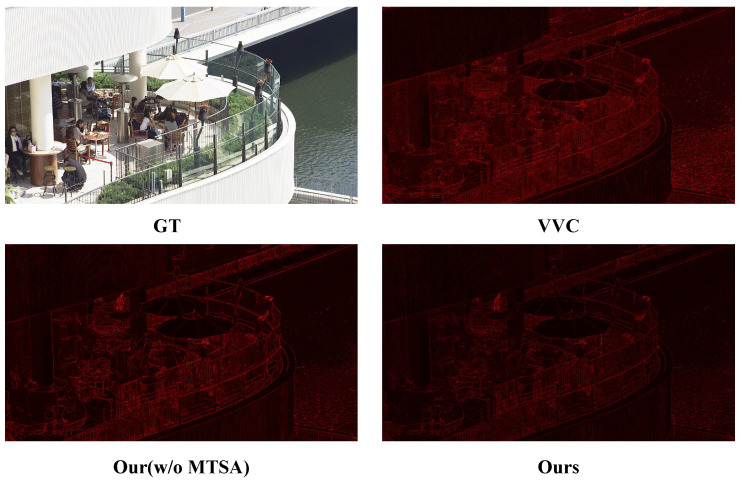
Residual heatmaps with respect to the ground truth; red regions indicate higher reconstruction errors.

To further validate the advantages of the MTSA module, Figure 7 shows the residual error maps between each reconstruction method and the ground truth (GT). All residuals are visualized using a heatmap, where red indicates higher pixel-wise errors. The top-left shows the original GT image, while the top-right presents the result from VVC compression. The bottom-left and bottom-right display our full model and the ablated version without the MTSA module (ours w/o MTSA), respectively. It can be observed that the full model preserves more structure and semantics with lower residuals, especially around object boundaries.

**Figure 8:**
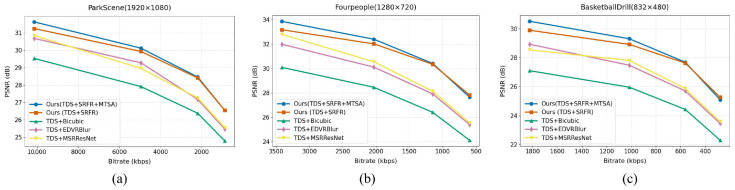
Ablation study of our proposed model. Rate–distortion curves on three video sequences comparing different video super-resolution methods. The full model (TDS + SRFR + MTSA) consistently achieves the best performance across all bitrates. Results are reported for (**a**) ParkScene (1920 × 1080); (**b**) Fourpeople (1280 × 720); and (**c**) BasketballDrill (832 × 480).

**Figure 9:**
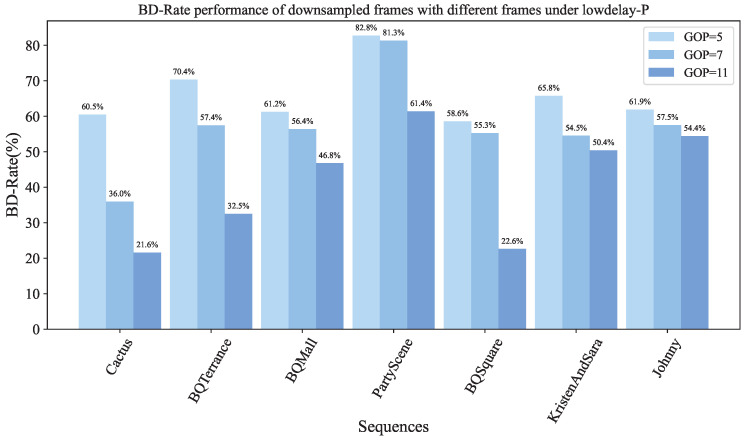
The histogram shows the changes in BD-rate of down-sampled frames between VVC and our proposed module at different number of frames (GOP = 5, 7, 11). The following comparisons are all performed under LDP configuration.

(b) The effectiveness of integrating the MTSA module into the SRFR framework: To validate the effectiveness of integrating the MTSA module into our SRFR framework, we conduct comprehensive ablation experiments across three representative video sequences: ParkScene, Fourpeople, and BasketballDrill. As shown in Figure 8, our baseline TDS + SRFR already delivers strong performance and significantly outperforms conventional enhancement methods such as Bicubic, MSRResNet, and EDVRBlur across all bitrate ranges. This confirms that the SRFR module itself is highly effective in restoring high-frequency details under compressed conditions. Building upon this strong baseline, incorporating MTSA yields further substantial gains. The full model TDS + SRFR + MTSA consistently achieves the highest PSNR on all three sequences and across all bitrate levels.

(c) BD-rate for down-sampled frames at different GOP sizes: To observe the impact of different GOPs on the results, we conduct experiments under different QP settings across multiple sequences, and evaluate segments containing 5, 7, and 11 frames. The downscaling ratio d is set to 4. Under the LDP configuration, we vary the GOP (Group of Pictures) size to analyze the influence of GOP length on the BD-rate of the downsampled frames. As shown in Figure 9, the histogram illustrates the BD-rate variations for GOP sizes of 5, 7, and 11 and compares the downsampled frames produced by our method with the original-size frames generated by standard VVC encoding. The experimental results show a clear trend: as the number of frames in the GOP increases, the temporal correlation between adjacent high-resolution reference frames gradually weakens, leading to a reduced BD-rate improvement for the downsampled frames.

Overall, the ablation results demonstrate that the integration of MTSA consistently improves both compression efficiency and reconstruction quality across all HEVC configurations. The performance gains stem from the structural design of MTSA, where the Multi-Temporal Attention (MTA) adaptively aggregates informative temporal references, and the Pyramid Spatial Attention (PSA) refines structural details through multi-scale spatial emphasis. Their complementary interaction enhances temporal coherence and spatial fidelity, while SRFR provides a strong reconstruction foundation. Together, these components enable stable and superior performance under both high- and low-bitrate conditions.

## 5. Discussion on Applications in Sensor-Based Systems

Modern sensing systems, including surveillance cameras, autonomous driving platforms, unmanned aerial vehicles (UAVs), mobile visual sensors, and Internet-of-Things (IoT) vision nodes, continuously generate large volumes of high-resolution video data. In many practical scenarios, these sensing devices operate under strict constraints on communication bandwidth, storage capacity, and computational resources. Efficient transmission of video streams from distributed sensor nodes to cloud or edge servers therefore becomes a critical challenge in intelligent sensing infrastructures.

The proposed temporal down-sampling-based compression framework is particularly suitable for such sensor-oriented applications. By selectively preserving high-quality boundary frames while aggressively compressing intermediate frames, the framework significantly reduces bitrate while maintaining essential semantic and structural information. This design aligns well with large-scale sensor networks, where continuous monitoring is required but full-resolution transmission of every frame is unnecessary.

Furthermore, many intelligent sensing systems rely on downstream machine vision tasks, such as object detection, multi-object tracking, anomaly recognition, and scene understanding. Our framework explicitly preserves critical spatial and temporal cues during compression, ensuring that reconstructed video streams remain suitable for both human observation and automated machine analysis. This dual objective of visual fidelity and semantic integrity makes the proposed method attractive for smart surveillance systems, intelligent transportation systems, industrial inspection platforms, and remote sensing applications. From a system-level perspective, the proposed method is codec-agnostic and can be seamlessly integrated into existing HEVC/VVC-based sensor pipelines without modifying the standard encoder or decoder structures. This compatibility facilitates practical deployment in real-world sensing infrastructures while preserving standard compliance and hardware implementation stability.

In summary, the proposed framework provides an effective solution for bandwidth-efficient and semantically robust video transmission in modern sensor-based visual systems, contributing to intelligent sensing, edge computing, and large-scale distributed monitoring applications.

## 6. Conclusions

This paper proposes a temporal down-sampling-based video coding framework (TDS) compatible with HEVC and VVC standards to prevent the loss of important information. To enhance down-sampled frames, we adopt a SR-based Frame Recurrent (SRFR) method that effectively leverages temporal information from neighboring high-resolution frames. Additionally, a Multi-Scale Temporal-Spatial Attention (MTSA) module is introduced to improve feature fusion and restoration accuracy. Under 2× downsampling, we observe BD-rate reductions of I, P, and B frames ranging from 14% to 39% under three configurations. Under 4× downsampling, we test the variations in BD-rate for down-sampled frames when comparing the VVC standard at different GOP sizes. The results show that our method achieves the best BD-rate reduction on downsampled frames at GOP = 5. Furthermore, our method achieves superior BD-rate performance compared to many prior works when anchored to the standard HEVC. Additionally, experiments show that our model exhibits strong adaptability and versatility across different scenarios, further validating the effectiveness of the proposed approach.

## Figures and Tables

**Table 1:** Objective comparison between the standard VVC and our proposed method using LDP, AI and RA. This work outperforms VVC in the average BD-rate, BD-psnr. The table shows the results of the calculations for P/B/I frames under GOP = 5. And the downsampling rate is 2.

Class	Sequence	Low Delay P		Random Access		All Intra	
		P Frames		B Frames		I Frames	
		BD-Rate	BD-PSNR	BD-Rate	BD-PSNR	BD-Rate	BD-PSNR
B	Kimono	−25.543	0.607	−39.803	0.909	−13.964	0.377
	ParkScene	−35.154	0.742	−33.141	0.643	−33.415	0.896
	BQTerrace	−24.129	0.675	−23.684	0.520	−27.364	0.842
	Cactus	−27.992	0.918	−33.164	0.474	−26.592	0.759
C	BasketballDrill	−35.807	0.908	−23.090	0.270	−23.236	0.718
	BQMall	−17.490	0.304	−26.226	0.476	−21.593	0.666
	PartyScene	−21.597	0.410	−27.369	0.238	−21.225	0.530
E	Johnny	−25.451	1.047	−22.865	0.870	−25.731	1.038
	KristenAndSara	−13.922	0.503	−20.632	0.242	−25.655	1.095
	Fourpeople	−24.566	0.810	−22.010	0.813	−30.066	1.357
Avg on Class B		−28.205	0.736	−32.448	0.637	−25.334	0.719
Avg on Class C		−24.965	0.541	−25.562	0.328	−22.018	0.638
Avg on Class E		−21.313	0.787	−21.836	0.642	−27.151	1.163
Average		−25.165	0.692	−27.198	0.546	−24.884	0.828

**Table 2:** Comparison of BD-Rate reduction and BD-PSNR between our method and Wu [59] under VTM’s AI configuration. The bold values represent the best results among the compared method.

Sequence	Wu [59]		Ours	
	BD-Rate	BD-PSNR	BD-Rate	BD-PSNR
Kimono	−7.69	0.40	−13.964	0.377
ParkScene	−4.24	0.13	−33.415	0.896
BQTerrace	−6.65	0.26	−27.364	0.842
Cactus	−10.3	0.41	−26.592	0.759
BasketballDrill	−11.32	0.20	−23.236	0.718
BQMall	1.14	−0.11	−21.593	0.666
PartyScene	3.58	−0.12	−21.225	0.530
Johnny	−8.80	0.44	−25.731	1.038
KristenAndSara	−7.72	0.43	−25.655	1.095
Fourpeople	−6.99	0.37	−30.066	1.357
Average	−5.899	0.241	**−24.884**	**0.828**

**Table 3:** The BD-rate reduction (%) comparison of test sequences for AI configuration on HM16.5. The red ones are the best results, and the blue ones are the second best results.

Class	Sequence	DCTIF [61]	Li [62]	Li + CNN [63]	Son [64]	Jiang [50]	Wei [51]	Chen [52]	Wu [59]	Our (w/o MTSA)	Our
B	Kimono	−3.40	−7.70	−9.00	−11.70	−2.83	−13.11	−12.41	−17.04	−31.733	−30.091
	ParkScene	−5.00	−7.10	−8.30	−11.60	−8.42	−12.67	−10.73	−15.22	−38.502	−39.574
	BQTerrace	−3.10	−3.70	−4.80	−14.10	−5.81	−	−7.63	−22.25	−29.975	−34.511
	Cactus	−6.60	−5.00	−8.50	−14.90	−10.28	−18.92	−13.22	−21.56	−29.977	−31.016
C	BasketballDrill	−4.90	−4.00	−7.50	−19.20	−17.87	−18.09	−19.04	−27.37	−30.456	−32.246
	BQMall	−2.30	−2.90	−3.90	−7.30	−14.32	−5.84	−14.47	−14.69	−26.912	−28.272
	PartyScene	−1.00	−1.00	−1.90	−9.30	−11.32	−8.36	−12.96	−14.89	−30.953	−28.244
E	Johnny	−9.00	−7.10	−10.20	−20.90	−7.32	−13.30	−11.18	−22.80	−31.464	−32.624
	KristenAndSara	−6.30	−5.80	−7.90	−15.70	−11.53	−7.98	−13.15	−21.71	−23.184	−27.226
	Fourpeople	−7.20	−7.20	−9.10	−15.20	−14.91	−10.84	−15.20	−20.01	−20.389	−26.535
Avg on Class B		−4.525	−5.875	−7.650	−13.075	−6.835	−14.900	−10.998	−19.018	−32.547	−33.798
Avg on Class C		−2.733	−2.633	−4.433	−11.933	−14.503	−10.763	−15.490	−18.983	−29.440	−29.587
Avg on Class E		−7.500	−6.700	−9.067	−17.267	−11.253	−10.707	−13.177	−21.507	−25.012	−28.795
Average		−4.880	−5.150	−7.110	−13.990	−10.461	−12.123	−12.999	−19.754	−29.355	−31.034

**Table 4:** Objective comparison between our proposed method and RR-DnCNN, RR-DnCNN-V2 using LDP and RA based on HEVC (HM16.5) in QPs = {32, 37, 42, 47}. This work outperforms the other two works in average BD-rate and BD-PSNR. The table shows results for all frames under GOP = 5.

Comparison of BD-Rate and BD-PSNR Gains Between Our Work and Two Other Works										
Size		Sequence	LDP				RA			
			RR-DnCNN		RR-DnCNN V2.0		RR-DnCNN		RR-DnCNN V2.0	
			BD-PSNR	BD-Rate	BD-PSNR	BD-Rate	BD-PSNR	BD-Rate	BD-PSNR	BD-Rate
Class B	1920 × 1072	Kimono	0.731	−21.927	0.663	−19.972	0.315	−10.184	0.237	−7.963
		ParkScene	0.385	−16.983	0.192	−8.651	0.277	−12.401	0.092	−3.819
		BQTerrace	0.084	−8.124	0.005	−4.288	0.106	−5.812	0.011	−1.347
		Cactus	0.673	−25.121	0.244	−9.061	0.424	−17.501	0.026	−1.566
Class C	832 × 480	BasketballDrill	0.037	−1.070	−0.028	0.938	0.667	−19.778	0.611	−18.244
		BQMall	−0.178	6.311	−0.192	6.829	0.023	−0.802	0.017	−0.633
		PartyScene	0.178	−12.793	0.190	−13.116	−0.120	6.191	−0.093	4.713
Class E	1280 × 720	Johnny	0.090	−2.458	−0.078	1.941	0.479	−14.058	−0.068	2.046
		KristenAndSara	0.173	−4.879	0.661	−16.355	−0.494	13.734	0.025	−5.831
		Fourpeople	0.701	−19.897	0.190	−7.604	0.372	−12.176	−0.131	4.691
Avg on Class B			0.468	−18.037	0.276	−10.493	0.281	−11.475	0.092	−3.674
Avg on Class C			0.0123	−2.517	−0.010	−1.783	0.190	−4.769	0.178	−4.721
Avg on Class E			0.3213	−9.078	0.2576	−7.3393	0.119	−4.167	−0.058	0.302
Average			0.287	−10.694	0.185	−6.934	0.205	−7.279	0.073	−2.795

**Table 5:** The comparison of parameters, running GPU memory and BD-rate (HM16.5 AI configuration) among six methods. The bold values indicate the lowest parameter count, the lowest GPU memory usage, and the best BD-rate performance among the compared methods.

Methods	Parameters (M)	GPU (M)	BD-Rate (%)
Li [62]	**0.396**	-	−5.15
Li + CNN [63]	0.693	**1207**	−7.11
Chen [52]	2.662	7398	−12.999
Jiang [50]	40.954	4306	−10.461
Wu [59]	1.862	3404	−19.754
Our (w/o MTSA)	24.7	2232	−29.36
Our	29.3	4084	**−31.03**

**Table 6:** Ablation study of our different models based on VTM. The bold values represent the best results among the compared method.

Model	Low Delay P		Random Access		All Intra	
	BD-Rate (%)	BD-PSNR (%)	BD-Rate (%)	BD-PSNR (%)	BD-Rate (%)	BD-PSNR (%)
TDS + SRFR	−20.779	0.446	−18.882	0.236	−20.372	0.534
TDS + SRFR + MTSA	**−25.165**	**0.692**	**−27.198**	**0.546**	**−24.884**	**0.828**

**Table 7:** Ablation study of our different models using AI configuration based on HEVC. The bold values represent the best results among the compared method.

Methods	BD-PSNR	BD-Rate
TDS + SRFR	0.856	−29.355
TDS + SRFR + MTSA	**1.157**	**−31.034**

**Table 8:** The ablation study and objective comparison between our proposed method and RR-DnCNN, RR-DnCNN-V2 using LDP and RA configurations based on HEVC. The bold values represent the best results among the compared method.

Model	Low Delay P				Random Access			
	RR-DnCNN		RR-DnCNN V2.0		RR-DnCNN		RR-DnCNN V2.0	
	BD-PSNR	BD-Rate	BD-PSNR	BD-Rate	BD-PSNR	BD-Rate	BD-PSNR	BD-Rate
TDS + SRFR	0.163	−6.243	0.069	−2.667	0.144	−5.309	0.015	−0.511
TDS + SRFR + MTSA	**0.287**	**−10.694**	**0.185**	**−6.934**	**0.205**	**−7.279**	**0.073**	**−2.795**

## Data Availability

The original contributions presented in this study are included in the article. Further inquiries can be directed to the corresponding author.

## Abbreviations

The following abbreviations are used in this manuscript:

VVC	Versatile Video Coding
HEVC	High Efficiency Video Coding
LR	Low Resolution
HR	High Resolution
AI	All Intra
LDP	Low Delay-P
RA	Random Access
QP	Quantization Parameter
GOP	Group of Pictures
BD-rate	Bjntegaard Delta rate
PSNR	Peak Signal-to-Noise Ratio
GT	Ground Truth
